# Inhibitory Effects of Constituents from *Morus alba* var. *multicaulis* on Differentiation of 3T3-L1 Cells and Nitric Oxide Production in RAW264.7 Cells

**DOI:** 10.3390/molecules16076010

**Published:** 2011-07-19

**Authors:** Zhi-Gang Yang, Keiichi Matsuzaki, Satoshi Takamatsu, Susumu Kitanaka

**Affiliations:** School of Pharmacy, Nihon University, 7-7-1 Narashinodai, Funabashi, Chiba 274-8555, Japan; E-Mails: yang_zhg@hotmail.com (Z.-G.Y); matsuzaki.keiichi@nihon-u.ac.jp (K.M.); takamatsu.satoshi@nihon-u.ac.jp (S.T.)

**Keywords:** *Morus alba* var. *multicaulis* Perro, arylbenzofuran, prenyl-flavonoid, adipocyte, macrophage

## Abstract

A new arylbenzofuran, 3′,5′-dihydroxy-6-methoxy-7-prenyl-2-arylbenzofuran (**1**), and 25 known compounds, including moracin R (**2**), moracin C (**3**), moracin O (**4**), moracin P (**5**), artoindonesianin O (**6**), moracin D (**7**), alabafuran A (**8**), mulberrofuran L (**9**), mulberrofuran Y (**10**), kuwanon A (**11**), kuwanon C (**12**), kuwanon T (**13**), morusin (**14**), kuwanon E (**15**), sanggenon F (**16**), betulinic acid (**17**), uvaol (**18**), ursolic acid (**19**), *β*-sitosterol (**20**), oxyresveratrol 2-*O*-*β*-d-glucopyranoside (**21**), mulberroside A (**22**), mulberroside B (**23**), 5,7-dihydroxycoumarin 7-*O*-*β*-d-glucopyranoside (**24**), 5,7-dihydroxycoumarin 7-*O*-*β*-d-apiofuranosyl-(1→6)-*O*-*β*-d-glucopyranoside (**25**) and adenosine (**26**), were isolated from *Morus alba* var. *multicaulis* Perro. (Moraceae). Their structures were determined by spectroscopic methods. The prenyl-flavonoids **11**–**14**, **16**, triterpenoids **17**,**18** and **20** showed significant inhibitory activity towards the differentiation of 3T3-L1 adipocytes. The arylbenzofurans **1**–**10** and prenyl-flavonoids **11**–**16** also showed significant nitric oxide (NO) production inhibitory effects in RAW264.7 cells.

## 1. Introduction

Obesity has become a global epidemic in both developed and developing countries, and it is often accompanied by hyperglycemia, hypertension, and hyperlipidemia, which together are called metabolic syndrome [1]. Adipose tissue is composed of various cell types; mature adipocytes, preadipocytes, fibroblasts, endothelial cells, vascular cells, and macrophages [2]. Obesity is a condition in which adipocytes accumulate a large amount of lipids and become enlarged. It is characterized at the cellular level by an increase in the number and size of adipocytes differentiated from fibroblastic preadipocytes in adipose tissue [3]. The cytosolic enzyme glycerol-3-phosphate dehydrogenase (GPDH) appears to have an important role in the conversion of glycerol into triglyceride (TG), and the level of activity of GPDH increases during the conversion of 3T3 cells [4]. Recent studies indicate that obesity is associated with low-grade chronic inflammation of adipose tissues, and that such inflammation is one of the potential mechanisms leading to insulin resistance [5]. It has been demonstrated that obese adipose tissue is characterized by increased infiltration of macrophages. In a coculture system of 3T3-L1 adipocytes and RAW264 macrophages, marked increases in secretion levels of inflammatory mediators such as TNF-α, MCP-1, and NO were observed [6,7]. NO is a short-lived free radical produced from L-arginine by nitric oxide synthase (NOS). NO mediates diverse functions by acting on various cells through interactions with different molecular targets, and excessive NO production is involved in various types of inflammation [8]. Therefore, we attempted to identify natural anti-inflammatory compounds that not only inhibit triglyceride accumulation by 3T3-L1 adipocytes, but also inhibit the secretion of NO from RAW 264.7 cells.

*Morus* (Moraceae), more commonly known as mulberry, is widely distributed throughout Asia, Europe, North and South America, and Africa, and is an economically important plant used for sericulture in East, Central and South Asia. This genus contains a variety of phenolic compounds including isoprenylated flavonoids, 2-arylbenzopyrans, stilbenes, coumarins, and Diels-Alder adduct compounds [9,10,11,12,13,14]. Almost all parts of *M. alba* L. have been used as traditional Chinese medicine, the root bark called “Sang-Bai-Pi” in China, has long been used for removing heat from the lung, relieving asthma, and inducing dieresis [15]. This and other species of *Morus* have been reported to have antibacterial, antioxidative, antihypertensive, antihyperglycemic, antihyperlipidemic and aromatase inhibition activities [10,13,16,17,18,19,20,21,22]. 

**Figure 1:**
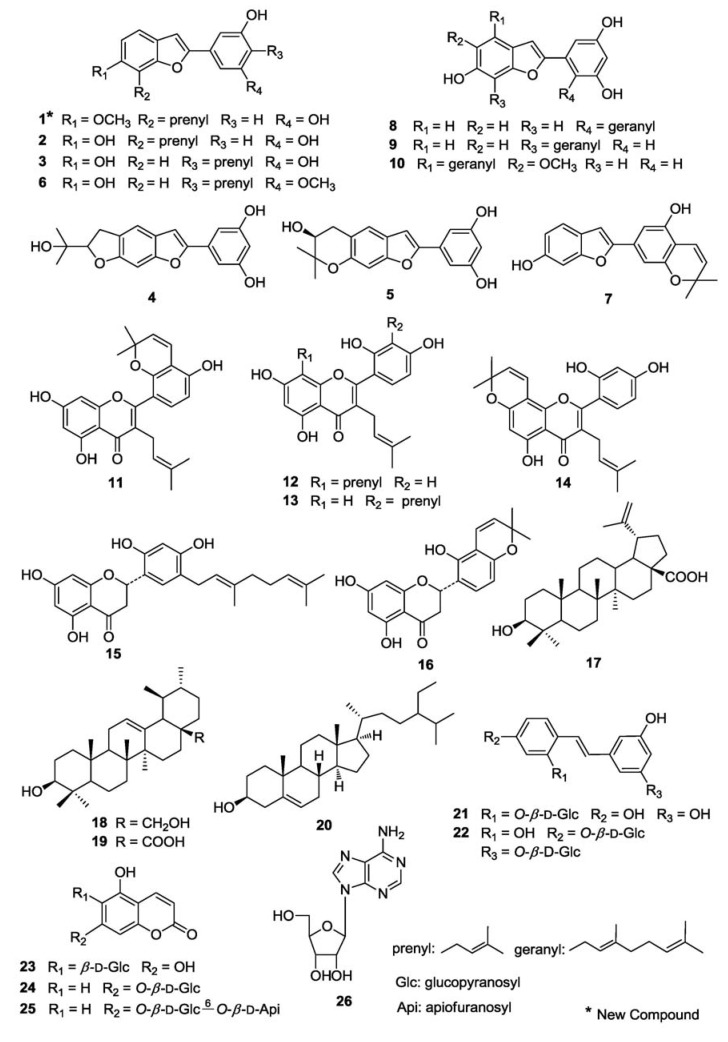
Structures of Compounds **1**–**2.**

In the present study, 80% aqueous methanol extracts of the root bark of *Morus alba* var. *multicaulis* Perro and the EtOAc-soluble portion of the extracts were observed to inhibit the accumulation of TG in 3T3-L1 cells, and inhibit NO production in lipopolysaccharide (LPS) and interferon-*γ* (IFN-*γ*) activated macrophages. Furthermore, a search for bioactive compounds present in this plant led to the isolation of a new compound, 3′,5′-dihydroxy-6-methoxy-7-prenyl-2-arylbenzofuran (**1**), and 25 known constituents: moracin R (**2**) [13], moracin C (**3**) [11], moracin O (**4**) [12], moracin P (**5**) [12], artoindonesianin O (**6**) [23], moracin D (**7**) [11], alabafuran A (**8**) [24], mulberrofuran L (**9**) [25], mulberrofuran Y (**10**) [26], kuwanon A (**11**) [27], kuwanon C (**12**) [27], kuwanon T (**13**) [28], morusin (**14**) [29], kuwanon E (**15**) [30], sanggenon F (**16**) [31], betulinic acid (**17**) [32], uvaol (**18**) [33], ursolic acid (**19**) [10], *β*-sitosterol (**20**) [10], oxyresveratrol 2-*O*-*β*-d-glucopyranoside (**21**) [34], mulberroside A (**22**) [35], mulberroside B (**23**) [36], 5,7-dihydroxycoumarin 7-*O*-*β*-d-glucopyranoside (**24**) [37], 5,7-dihydroxycoumarin 7-*O*-*β*-d-apiofuranosyl-(1→6)-*O*-*β*-d-glucopyranoside (**25**) [38] and adenosine (**26**) [39] (Figure 1). We also describe their inhibitory activity towards adipogenesis in 3T3-L1 adipocytes, and their inhibition of NO production in RAW 264.7 cells in this contribution. 

## 2. Results and Discussion

The root barks of *M. alba* var. *multicaulis* Perro. were extracted with 80% aqueous methanol and the solvent evaporated from the combined extract under reduced pressure to give the 80% methanol extract. The extract [TG and NO inhibitory activity: 23%, 72%, respectively (100 μg/mL)] was dissolved in water and successively partitioned into a chloroform layer [(46%, 96% (30 μg/mL)], ethyl acetate layer [(52%, 98% (30 μg/mL)], *n*-butanol layer [(45%, 28% (30 μg/mL)], and an aqueous fraction [(22%, 11% (30 μg/mL)]. Therefore, we have focused our efforts on researching the chemical constituents of the ethyl acetate soluble fraction, which was separated by silica gel column chromatography to yield 11 fractions. Twenty one compounds were isolated from these fractions. Five compounds were isolated from the *n*-butanol layer.

Compound **1** was obtained as a yellow amorphous powder. The molecular formula of **1** was established as C_20_H_20_O_4_ by HR-EI-MS. The UV spectrum exhibited absorption maxima at 216, 318, and 330 nm, suggesting the presence of a 2-arylbenzofuran skeleton [13]. The ^1^H-NMR spectrum of **1** indicated *ortho*-coupled proton signals at δ 7.38 (1H, d, *J* = 8.5 Hz) and δ 6.97 (1H, d, *J* = 8.5 Hz), and 1,3,5-distributed benzene signals at δ 6.91 (2H, d, *J* = 2.4 Hz) and δ 6.39 (1H, t, *J* = 2.4 Hz), and one proton of furan nucleus signal at δ 7.06 (1H, s), and prenyl proton signals at δ 5.38 (1H, m), δ 3.63 (2H, d, *J* = 7.3 Hz), δ 1.88 (3H, s) and δ 1.67 (3H, s). The spectrum also showed one methoxy proton signal at δ 3.90 (3H, s). The ^1^H-NMR spectrum of **1** was similar to that of **2**, except for the methoxy proton signal (Table 1).

**Figure 2:**
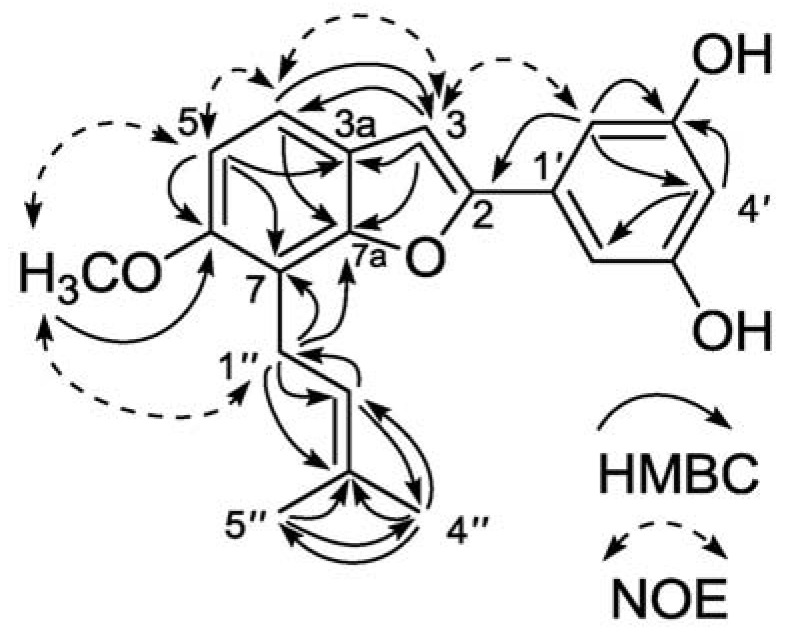
Key HMBC and NOE correlations of **1**.

The ^13^C-NMR and distortionless enhancement by polarization transfer (DEPT) spectra revealed the presence of two methyls (δ 18.0, 25.9), one methoxy (δ 56.9), one methylene (δ 23.4), seven methines [δ 102.4, 103.7, 103.9 (×2), 109.0, 119.0, 123.0], and nine quaternary carbons [δ 113.9, 123.8, 132.1, 133.4, 154.9, 156.0, 156.1, 159.8 (×2)]. From the HMBC (Figure 2) spectrum, the methylene proton δ 3.63 (H-1″) showed long-range correlation with δ 113.9 (C-7) and δ 154.9 (C-7a); the methoxyl proton δ 3.90 with δ 156.0 (C-6); and δ 6.97 (H-5) with δ 113.9 (C-7), δ 123.8 (C-3a) and δ 156.0 (C-6), indicating that the location of the prenyl group was at C-7, and the methoxyl group at C-6. Furthermore, in the elected NOE (Figure 2) spectrum, correlations were observed between δ 3.63 (H-1″) /δ 3.90 (OMe), δ 6.97 (H-5) /δ 3.90, δ 6.97/δ 7.38(H-4), δ 7.38/δ 7.06(H-3), δ 7.06/δ 6.91(H-2′, 6′). Thus, the structure of **1** was determined as 3′, 5′-dihydroxy-6-methoxy-7-prenyl-2-arylbenzofuran. 

In addition, compounds **2**–**26** were identified as known compounds by detailed comparisons of their spectroscopic data with that in the literature. 

Compounds **1**–**26** were evaluated for their inhibitory effects on triglyceride accumulation and GPDH activity in 3T3-L1 cells at the concentration of 20 μM (Table 2). Quercetin, which has been reported to have inhibitory effects on triglyceride accumulation, was used as a positive control [40]. No compound, except for **19**, exhibited cytotoxic effects in 3T3-L1 cells based on microscopic observation, furthermore, the percentage of survival cells examined by DNA quantity assays, and the cell viability of **19** was near 10%. As shown in Table 1, the prenyl-flavonoids **11**–**14**,**16**, triterpenoids **17**,**18** and *β*–sitosterol showed significant inhibition of adipogenesis in 3T3-L1 adipocytes, with TG inhibition values of 47.1%, 40.0%, 39.9%, 51.6%, 36.0%, 56.0%, 43.2% and 34.5%, respectively, which were stronger than the positive control, and quercetin whose TG inhibition was 34%. The flavones, **12** and **14** showed particularly strong inhibition of GPDH activity (60.1% and 70.0% inhibition, respectively), the flavanones, **15** and **16** showed weaker inhibitory effects of GPDH activity (12.0% and 18.1% inhibition, respectively) than other flavones. These results indicate that the prenyl moiety at C-3 and A-ring may have enhanced the anti-differentiation activities of 3T3-L1 cells. The 2-arylbenzofurans showed moderate or weak inhibitory effects on TG and GPDH activity. Comparing **1** with **2**, **3** with **6** and **9** with **10**, compounds **1**, **6** and **10** showed stronger TG and GPDH activity inhibition than **2**, **3** and **9**, respectively, these results suggest that the methoxy group may enhance their activities. The major component, mulberroside A (**22**, 0.23% yield), showed 16.7% TG inhibition and 22.8% GPDH activity inhibition.

**Figure 3:**
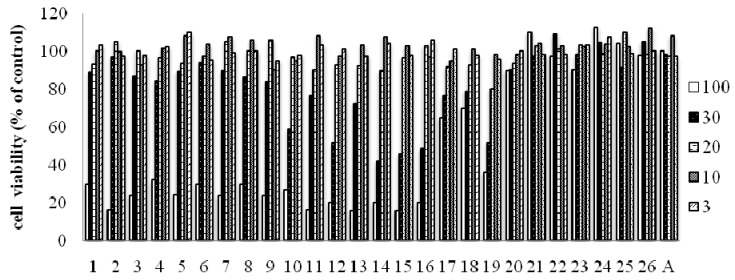
Effects of isolated compounds on cell viability of RAW264.7 cells by MTT assay.

Notes: RAW264.7 cells were treated with isolated compounds at indicated concentrations (100, 30, 20, 10, 3 μM, respectively), cell viability was measured by the 3-(4,5-dimethyl-2-thiazolyl)-2,5-diphenyl-2H-tetrazolium bromide (MTT) assay. Data are expressed as a percentage of the control levels; A: aminoguanidine.

It has been demonstrated that macrophages treated with LPS and/or IFN-*γ*, increase secretion of several cytokines. LPS and IFN-*γ* responsive pathways in macrophages are distinct and independent, and these two inducers can cooperate with each other in achieving full macrophage activation [41,42]. Macrophages, treated with LPS and IFN-*γ*, produce 5–6 fold higher amounts of NO than with LPS alone [43]. Therefore, compounds **1**-**26** were also examined with respect to their inhibition of NO production stimulated by LPS and IFN-*γ* in RAW 264.7 cells (Table 3). In the assay, aminoguanidine (IC_50_ 17.5 μM), which has been reported to have inhibitory effects on NO production in LPS activated RAW 264.7 macrophages via the down-regulation of inducible nitric oxide synthase (iNOS), was used as a positive control [44]. As shown in Table 3, most of the arylbenzofurans (compounds **1**–**10**) and prenyl-flavonoids (compounds **11**–**16**) showed strong or moderate inhibitory effects on NO production compared with aminoguanidine. The triterpenoids **17** and **19** also exhibited weak or strong NO production inhibitory activity; however, **18** did not show such activity may because of a lack of the carbonyl group at C-28. Furthermore, effects of isolated compounds on cell viability of RAW264.7 cells were measured by MTT assay. As shown in Figure 3, the isolated compounds had no effects on viability of RAW264.7 at concentration of IC_50_ values.

## 3. Experimental 

### 3.1. General

UV spectra were obtained in MeOH on a Shimadzu UV-160 spectrophotometer. The NMR spectra were recorded on a JEOL ECA 600 MHz spectrometer, with TMS as an internal standard. The MS were obtained on a JEOL GCmate spectrometer. Column chromatography was carried out with Silica gel 60N (KANTO Chemical CO., INC.), Sephadex LH-20 (Pharmacia), Diaion HP-20 (Nippon Rensui) and Chromatorex ODS (Fuji Silysia Chemical Ltd.). Thin-layer chromatography (TLC) was performed on Merck TLC plates (0.25 mm thickness), with compounds visualized by spraying with 5% (v/v) H_2_SO_4_ in ethanol solution and then heating on a hot plate. HPLC was performed on a JASCO PU-2089 apparatus equipped with JASCO UV-2075. Shiseido SG80A (10 × 150 mm i.d.), Cosmosil 5C18 PAQ (10 × 150 mm i.d.) and Shiseido CAPCELL PAK C18 (10 × 150 mm i.d.) were used for preparative purposes. 

### 3.2. Plant Material

The root barks of *M. alba* var. *multicaulis* Perro. were collected in Chiba, Japan, in March 2007 and were identified by Dr. Heran Li of the College of Pharmaceutical Science, Soochow University, People’s Republic of China. Voucher specimens have been deposited at the Laboratory of Pharmacognosy, School of Pharmacy, Nihon University.

### 3.3. Extraction and Isolation

The root barks of *M. alba* var. *multicaulis* Perro. (9.0 kg) were extracted three times with 80% methanol and concentrated to give the methanol extract. The extract (1311 g) was dissolved and suspended in water (6 L) and partitioned to form a chloroform layer (8.7 g), ethyl acetate layer (356.9 g), *n*-butanol layer (225.0 g) and water layer (758.3 g).

The ethyl acetate layer was subjected to silica gel column chromatography and eluted with CHCl_3_–CH_3_OH to afford 11 fractions (Fr.1-11). Fraction 9 was purified using ODS column chromatography and eluted with CH_3_OH–H_2_O to give 12 fractions (Fr.9-1~12). Fraction 9-6 was purified using silica gel column chromatography, and reverse-phase HPLC eluted with CH_3_CN–H_2_O to give **2** (25 mg), **3** (30 mg), **4** (62 mg) and **5** (20 mg). Fraction 9-8 was chromatographed on a Sephadex LH-20 column to give 9 fractions (Fr.9-8-1~9). Fraction 9-8-4 was purified using a silica gel column and reverse-phase HPLC, and eluted with CH_3_OH–H_2_O to give **1** (5 mg), **6** (4 mg), **11** (30 mg), **12** (35 mg), **13** (40 mg) and **16** (4 mg). Fraction 9-8-6 was chromatographed on an ODS column and normal-phase HPLC and eluted with *n*-hexane–EtOAc to give **7** (39 mg), **8** (45 mg), **9** (40 mg), **10** (15 mg) and **15** (430 mg). Fraction 5 was purified using silica gel column chromatography and eluted with *n*-hexane–EtOAc to give **14** (28 mg), **17** (220 mg), **18** (6 mg), **19** (7 mg) and **20** (210 mg). Fraction 10 was purified using HP-20, ODS and Sephadex LH-20 column chromatography to give **21** (56 mg).

The *n*-butanol layer was subjected to HP-20 column chromatography and eluted with CH_3_OH–H_2_O and 70% acetone to afford 4 fractions (Fr.1-4). Fraction 2 was purified using silica gel column chromatography and eluted with CHCl_3_–CH_3_OH and reverse-phase HPLC eluted with CH_3_OH–H_2_O to give 2**2** (21 g), **23** (5 mg), **24** (4 mg), **25** (51 mg) and **26** (10 mg). 

*3′, 5′-dihydroxy-6-methoxy-7-prenyl-2-arylbenzofuran* (**1**). Yellow amorphous powder; EI-MS (m/z): 324 [M]^+^, HR-EI-MS m/z: found. 324.1360 (calcd 324.1361 for C_20_H_20_O_4_); UV(MeOH) λmax(logε): 216 (4.31), 318 (4.20), 330 (4.17); ^1^H- and ^13^C-NMR (acetone-*d*_6_, 600/150MHz) data: See Table 1.

### 3.4. Triglyceride (TG) content and Glycerol-3-phosphate dehydrogenase (GPDH) activity in 3T3-L1 cells *[45]*

3T3-L1 preadipocytes (American Type Culture Collection, Manassas, VA, USA) were subcultured in Dulbecco’s Modified Eagle’s Medium (DMEM) containing 10% newborn calf serum (Gibco) at 37 °C under a humidified 5% CO_2_ atmosphere. Briefly, two days after reaching confluence (day 0), the 3T3-L1 preadipocytes were induced by switching the differentiation medium to DMEM containing 10% fetal bovine serum (FBS) (SAFC Biosciences), 500 μM 3-isobutyl-1-methylxanthine (Sigma), 1 μM dexamethasone (Sigma), and 10 μg/mL insulin (Sigma) for three days. The medium was then replaced with DMEM containing 10% FBS and 5 μg/mL insulin, and was changed every two or three days.

The 3T3-L1 preadipocytes were seeded at 1.0 × 10^5^ cells/mL onto 24-well plates (Sumitomo Bakelite, MS-80240, Tokyo) and incubated at 37 °C. A test sample was added to the medium on day 0, and added at the time of every medium change during the 8 days of incubation. After removing the medium, the cells were washed twice with 500 μL of PBS. The cells were collected in 500 μL of cold sonication buffer (pH 7.5 25 mM Tris buffer containing 1 mM EDTA) and sonicated in ice-cold water. After centrifugation, the cell lysate was used to measure the TG content with LabAssay^TM^ Triglyceride (WAKO Pure Chemical Industries Ltd.), GPDH activity with a GPDH Assay Kit (TaKaRa Bio Inc.), and DNA quantity with a DNA Quantity Kit (Primary Cell Co., Ltd.), according to the manufacturer’s protocol. Inhibition of TG and GPDH activity was calculated using the following formula: Inhibition (%) = [(*Cn*−*S*)/*Cn*] × 100, where *S* is TG amount or GPDH activity when cells incubated with sample and divided by the amount of DNA for each well; *Cn* is TG amount or GPDH activity when cells incubated with DMSO (control) were divided by the amount of DNA for each well). Cell viability was confirmed by microscopic observation.

### 3.5. NO Production in Activated Macrophage-Like Cell Line, RAW 264.73 *[46,47]*

The macrophage-like cell line, RAW 264.7, was obtained from American Type Culture Collection. The cells were cultured in Ham’s F12 medium with 10% FBS (SAFC Biosciences) at 37 °C under a humidified 5% CO_2_ atmosphere. The RAW264.7 cells were seeded at 1.2 × 10^6^ cells/mL onto 96-well plates (Sumitomo Bakelite, MS-8096R, Tokyo) and then incubated at 37 °C for 2 h. A test sample was then added to the culture simultaneously with both *Escherichia coli* LPS (100 ng/mL) and recombinant mouse IFN-γ (0.33 ng/mL), and the cells were incubated at 37 °C, usually for 16 h. The amount of nitrite in culture supernatants was measured using the Griess assay. Cell viability was measured using the 3-(4,5-dimethyl-2-thiazolyl)-2,5-diphenyl-2H-tetrazolium bromide (MTT) assay method. 

## 4. Conclusions

Obesity is associated with low-grade chronic inflammation of adipose tissues, and obese adipose tissue is characterized by increased infiltration of macrophages. The root bark extract of *Morus alba* var. *multicaulis* Perro. showed inhibitory effects on the differentiation of 3T3-L1 cells and NO production in RAW264.7 cells. Bioguided assay of bioactive constituents led to the identification of a new compound, 3′,5′-dihydroxy-6-methoxy-7-prenyl-2-arylbenzofuran (**1**) and 25 known constituents. The isolated compounds, especially the prenyl-flavonoids and 2-arylbenzofurans, suppressed adipogenesis in 3T3-L1 adipocytes and NO production in RAW264.7 cells. In conclusion, the findings of the present study may account for the use of *M. alba* var. *multicaulis* Perro. in traditional medicine to treat inflammation, and the isolated compounds might be a source of anti-obesity and anti-inflammatory agents to improve the symptoms of metabolic syndrome.

## Figures and Tables

**Table 1:** ^1^H- and ^13^C-NMR Spectral Data of **1**, **2** [(600/150 MHz, acetone-*d*_6_, TMS, *δ* (ppm) (*J* = Hz)].

	1		2	
position	*δ*_H_	*δ*_C_	*δ*_H_	*δ*_C_
2		156.1		154.6
3	7.06 (s)	102.4	7.02 (s)	101.8
3a		123.8		121.8
4	7.38 (d 8.5)	119.0	7.23 (d 8.5)	118.1
5	6.97 (d 8.5)	109.0	6.84 (d 8.5)	112.3
6		156.0		152.7
7		113.9		111.3
7a		154.9		154.5
1^′^		133.4		132.8
2^′^	6.91 (d 2.4)	103.9	6.90 (d 2.3)	103.0
3^′^		159.8		159.0
4^′^	6.39 (t 2.4)	103.7	6.28 (t 2.3)	102.7
5^′^		159.8		159.0
6^′^	6.91 (d 2.4)	103.9	6.90 (d 2.3)	103.0
1^″^	3.63 (d 7.3)	23.4	3.65 (d 7.5)	22.6
2^″^	5.38 (m)	123.0	5.45 (m)	122.3
3^″^		132.1		131.2
4^″^	1.67 (s)	25.9	1.67 (s)	25.1
5^″^	1.88 (s)	18.0	1.89 (s)	17.3
-OH	8.44(brs 2 x OH)		8.44(brs 2 x OH)	
-OCH_3_	3.90 (s)	56.9		

**Table 2:** Inhibition of TG Content and GPDH Activity by the isolated compounds in 3T3-L1 Adipocytes.

Compound	Inhibition (%)		Compound	Inhibition (%)	
	TG	GPDH activity		TG	GPDH activity
**1**	20.3 ± 2.1	26.2 ± 3.8	**14**	51.6 ± 2.7	70.0 ± 8.3
**2**	14.2 ± 2.0	11.9 ± 1.3	**15**	23.4 ± 3.8	12.0 ± 2.1
**3**	13.8 ± 3.1	8.2 ± 0.7	**16**	36.0 ± 8.0	18.1 ± 7.2
**4**	6.7 ± 1.2	18.0 ± 2.7	**17**	56.0 ± 4.7	44.1 ± 5.3
**5**	8.9 ± 2.4	19.0 ± 3.0	**18**	43.2 ± 6.1	20.2 ± 6.8
**6**	18.4 ± 4.2	10.7 ± 1.5	**19**	―	―
**7**	14.5 ± 2.0	12.9 ± 1.8	**20**	34.5 ± 8.8	26.9 ± 7.0
**8**	5.6 ± 1.8	4.0 ± 1.2	**21**	8.44 ± 2.0	10.5 ± 3.4
**9**	10.9 ± 2.5	19.4 ± 3.4	**22**	16.7 ± 7.8	22.8 ± 8.0
**10**	11.8 ± 1.3	25.9 ± 4.5	**23**	7.9 ± 1.2	5.2 ± 2.7
**11**	47.1 ± 5.2	38.9 ± 3.9	**24**	25.3 ± 7.5	9.2 ± 2.1
**12**	40.0 ± 3.0	60.1 ± 5.4	**25**	33.0 ± 2.3	13.9 ± 4.2
**13**	39.9 ± 3.9	28.7 ± 3.9	**26**	20.5 ± 4.5	8.0 ± 2.6
			Quercetin	33.8 ± 3.7	36.1 ± 4.0

Notes: 3T3-L1 adipocytes were harvested after 8 days of treatment with compound (20 μM) or vehicle, and assayed for total triglyceride (TG) content and glycerol-3-phosphate dehydrogenase (GPDH) activity. The reported values are the mean ± SD (*n* = 3); ―: Cytotoxic effects were observed at 20 μM.

**Table 3:** Inhibition by isolated compounds of NO production stimulated by LPS and IFN-γ in RAW 264.7 cells.

Compound	IC_50_ (μM)	Compound	IC_50_ (μM)
**1**	16.1	**15**	14.9
**2**	14.3	**16**	19.0
**3**	8.0	**17**	45.2
**4**	7.1	**18**	>100
**5**	19.3	**19**	17.8
**6**	18.7	**20**	>100
**7**	18.0	**21**	>100
**8**	10.2	**22**	>100
**9**	12.3	**23**	>100
**10**	9.2	**24**	>100
**11**	10.5	**25**	>100
**12**	12.6	**26**	>100
**13**	10.0	Aminoguanidine	17.5
**14**	10.6		
